# Proteomics Analysis of Cellular Proteins Co-Immunoprecipitated with Nucleoprotein of Influenza A Virus (H7N9)

**DOI:** 10.3390/ijms161125934

**Published:** 2015-10-30

**Authors:** Ningning Sun, Wanchun Sun, Shuiming Li, Jingbo Yang, Longfei Yang, Guihua Quan, Xiang Gao, Zijian Wang, Xin Cheng, Zehui Li, Qisheng Peng, Ning Liu

**Affiliations:** 1 Central Laboratory, Jilin University Second Hospital, Changchun 130041, China; sunnn13@mails.jlu.edu.cn (N.S.); yang.jb@live.cn (J.Y.); yanglongfei@jlu.edu.cn (L.Y.); qgh0521@jlu.edu.cn (G.Q.); gaoxiang13@mails.jlu.edu.cn (X.G.); jiuwzj1989@sina.com (Z.W.); chengxin14@mails.jlu.edu.cn (X.C.); lizh7013@mails.jlu.edu.cn (Z.L.); 2 Key Laboratory of Zoonosis Research, Ministry of Education, Institute of Zoonosis, Jilin University, Changchun 130062, China; Wanchunsun@jlu.edu.cn; 3 College of Life Sciences, Shenzhen University, Shenzhen 518057, China; shuimingli@szu.edu.cn

**Keywords:** nucleoprotein, influenza A virus, lysine acetylation, mass spectrometry, co-immunoprecipitation

## Abstract

Avian influenza A viruses are serious veterinary pathogens that normally circulate among avian populations, causing substantial economic impacts. Some strains of avian influenza A viruses, such as H5N1, H9N2, and recently reported H7N9, have been occasionally found to adapt to humans from other species. In order to replicate efficiently in the new host, influenza viruses have to interact with a variety of host factors. In the present study, H7N9 nucleoprotein was transfected into human HEK293T cells, followed by immunoprecipitated and analyzed by proteomics approaches. A series of host proteins co-immunoprecipitated were identified with high confidence, some of which were found to be acetylated at their lysine residues. Bioinformatics analysis revealed that spliceosome might be the most relevant pathway involved in host response to nucleoprotein expression, increasing our emerging knowledge of host proteins that might be involved in influenza virus replication activities.

## 1. Introduction

Avian influenza A viruses normally circulate among avian populations and do not efficiently infect humans. However, the viruses change constantly through genome mutation and reassortment and it is possible that these viruses could cross the species barrier to infect humans. The avian-origin influenza A virus strains such as H5N1 and H9N2 have shown their ability to cause severe infections in humans, including the 1997 and 2003 outbreaks in Hong Kong [1,2,3]. Recently, cases of human infections with newly reasserted avian influenza A (H7N9) virus have been continuously reported in China since March 2013 [4,5,6], which has received much attention as a potential pandemic threat to public health.

It has been well recognized that a virus, despite having few genes, utilizes many host factors for efficient viral replication in its host cell [7]. Therefore, it is very important to identify virus-host interactions as crucial determinations of host specificity, replication, and pathology. The genome of influenza A virus consists eight segmented negative-sense single-stranded RNA (vRNA), which is wrapped with viral nucleoprotein (NP). NP, one of major structural proteins in influenza virus, executes multiple functions necessary for replication and transcription of vRNA during the virus life cycle [8,9,10]. Many efforts have been made to search host proteins associated with NP, which play critical roles in assembling the viral RNA replication complex, recognizing viral RNA replication templates. The cytoskeleton scaffolding protein α-actinin-4 was identified as a novel interacting partner with influenza A viral NP in the virus infection period [11]. Using Gal4-based yeast two-hybrid (Y2H) assay, ten potential human host cell proteins that interact with influenza A viral NP were identified, which were involved in various host cell processes and structures [12]. In another work using strep-tagged viral nucleoprotein (NP-Strep) as bait, 41 vRNP-associated cellular interaction partners were identified by mass spectrometry [13]. In addition, the study of the interaction between host and virus can provide new ideas for the development of clinical drug target and the prevention of disease. For example, using forward chemical genetics, influenza A nucleoprotein (NP) was identified as a valid target for a compound, nucleozin, which could inhibit NP’s nuclear accumulation [14].

In the present study, we purified NP from transiently transfected HEK293T cells by co-immunoprecipitation, from which a series of host proteins co-immunoprecipitated were identified by proteomics approaches. Acetylation modifications on lysine residues of some host proteins were detected with high confidence. Bioinformatics analysis of the obtained proteomics data revealed that spliceosome might be the most relevant pathway involved in host response to nucleoprotein expression.

## 2. Results and Discussion

### 2.1. Expression and Immunoprecipitation of NP

**Figure 1:**
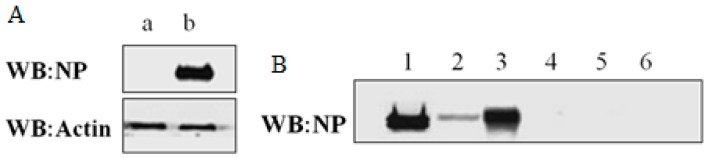
Western blot analysis of expression and immunoprecipitation of NP. (**A**) Over expression of NP in the HEK293T cells. β-actin was used as a loading control. a: empty pCMV; b: pCMV-NP; (**B**) Immunoprecipitation of NP.1 and 4 refer to lysates of cells transfected with pCMV-NP and those transfected with empty pCMV, respectively; 2 and 5 refer to supernatants of immunoprecipitated lysates 1 and 4, respectively; 3 and 6 refer to elutes from antibody-crosslinked beads that had been incubated with 1 and 4, respectively.

The pCMV-NP plasmid included full length NP sequence (Influenza A H7N9 (A/shanghai/1/2013)), which had Ampicillin resistance and thus could be purified and amplified in LB media supplemented with Ampicillin when transformed into E.coli DH5α. The sequence accuracy of cloned NP was confirmed by gene sequencing as indicated in Figure S1 in supplementary materials. The pCMV-NP plasmid was transfected into HEK293T cells and incubated for 36 h, whereas an empty pCMV plasmid was used as negative control in separate HEK293T cells. As expected, overexpression of NP was validated by western blotting assay, as shown in Figure 1A, in which parallel western blotting of actin was used as a protein loading quantification control. In immunoprecipitation experiments, a kit from Pierce was chosen. Of note, the kit enables highly efficient antigen immunoprecipitations by coupling antibody to the beads and then covalently crosslinking to the beads with DSS. As shown in Figure 1B, NP expressed in HEK293T cells transfected by pCMV-NP was efficiently immunoprecipitated using the crosslink IP kit.

### 2.2. Identification of Cellular Proteins Co-Immunoprecipitated with NP

The protein samples eluted from antibody-crosslinked beads by acidic Elution Buffer were neutralized and then buffer-exchanged with a buffer containing 8 M urea, followed by DTT reduction and IAA alkylation. The protein samples were then subject to tryptic digestion. The digests were analyzed by nano-LC-MS/MS, followed by protein identification through database searching. As expected, NP was solely identified in samples immunoprecipitated from pCMV-NP-transfected cells, of which 32 unique peptides were retrieved with sequence coverage (95) of 39.36% as indicated in Table S1 in supplementary materials.

Besides the identified NP, a series of cellular proteins were positively identified when the MS/MS data were searched against protein database of Homo sapiens instead of Influenza virus. These proteins were originated from host HEK293T cells and co-immunoprecipitated with NP. Notably, cellular proteins other than NP were detected in both samples from pCMV-NP-transfected cells and those from empty pCMV-transfected control cells. The proteins detected in the control sample were viewed as “background noise” signals, which were supposed to be resulted from non-specific binding with either NP or antibody-crosslinked beads or both. Therefore, proteins that were solely detected in co-immunoprecipitated samples from pCMV-NP-transfected cells but not from control cells were selected as identities specifically co-immunoprecipitated with NP, as indicated in Table 1. Additionally, proteins that were detected in co-immunoprecipitated samples from both pCMV-NP-transfected cells and control cells but had significantly higher summed peptide intensity (10-fold increase) in sample of pCMV-NP-transfected cells than in that of control cells were also included in Table 1. The detailed results from database searches were included into supplementary files (Table S2).

In our study, Co-IP followed by proteomics analysis was used to find proteins associated with H7N9 nucleoprotein, whereas most of similar published work used different approaches. For example, a yeast two-hybrid system was used to screen proteins interacted with NP [12]. Strep-tagged viral nucleoprotein (NP-Strep) was used to purify reconstituted vRNPs to identify cellular factors associated to these native viral complexes [13]. Different approaches usually resulted in some discrepancies between our work and other published studies. Nevertheless, some of results in our study are well validated by previously published work. Cytoskeleton scaffolding protein α-actinin-4 was found to be associated with H7N9 nucleoprotein in our study, which was previously identified as interacting partner with IAV nucleoprotein [11].

It should be noted that some well-established interacting partners IAV nucleoprotein such as importin and exportin were not detected in our assay. This might be due to the host restriction on the NP protein. It has been realized that NP not only displays a clear boundary between human and avian viruses from histogram analysis but also contains more species-associated amino acid signatures [15]. Within the NP, there are amino acid signatures found within different host species [16]. These host-specific amino acid residues may result in differences in affinities for the various host proteins with which they interact or they may result in differences in how the NP interacts with other viral proteins that have also made host-specific adaptations [17]. In our study, the NP was of typical avian origin, which might have too low affinities with some interacting partners in human cells to be captured by Co-IP.

**Table 1:** Identification of host cellular proteins co-immunoprecipitated with NP.

Accession Number	Names	Abbreviation	Average Mass (Da)	Theoretical pI	%Cov(95)	Number of Identified Peptides
P11940	Polyadenylate-binding protein 1	PABP1	70,670.84	9.52	39.15	29
Q59GN2	Putative 60S ribosomal protein L39-like 5	R39L5	6322.59	12.32	19.61	4
P02787	Serotransferrin	TRFE	77,063.89	6.81	41.83	31
Q12906	Interleukin enhancer-binding factor 3	ILF3	95,338.37	8.86	24.61	19
P09429	High mobility group protein B1	HMGB1	24,893.76	5.6	29.30	6
Q08211	ATP-dependent RNA helicase A	DHX9	140,958.5	6.41	28.90	31
P01024	Complement C3	CO3	187,148.1	6.02	19.78	28
Q00059	Transcription factor A, mitochondrial	TFAM	29,096.63	9.74	34.96	10
P26599	Polypyrimidine tract-binding protein 1	PTBP1	57,221.33	9.22	21.66	10
P25705	ATP synthase subunit α, mitochondrial	ATPA	59,750.63	9.16	21.88	9
P38159	RNA-binding motif protein, X chromosome	RBMX	42,331.85	10.06	38.11	14
P09874	Poly [ADP-ribose] polymerase 1	PARP1	113,083.8	8.99	18.05	15
P00738	Haptoglobin	HPT	45,205.31	6.13	34.24	12
P43243	Matrin-3	MATR3	94,623.24	5.87	20.78	18
P68032	Actin, α cardiac muscle 1	ACTC	42,018.97	5.23	43.24	11
P48681	Nestin	NEST	177,438.9	4.35	13.76	18
Q96PK6	RNA-binding protein 14	RBM14	69,491.65	9.68	16.59	10
Q13765	Nascent polypeptide-associated complex subunit α	NACA	23,383.9	4.52	26.76	4
Q15459	Splicing factor 3A subunit 1	SF3A1	88,886.18	5.15	3.03	2
Q14919	Dr1-associated corepressor	NC2A	22,349.84	5.04	31.60	6
P17844	Probable ATP-dependent RNA helicase DDX5	DDX5	69,148.08	9.06	14.50	9
P15927	Replication protein A 32 kDa subunit	RFA2	29,246.85	5.74	17.78	3
O75531	Barrier-to-autointegration factor	BAF	10,058.58	5.81	51.69	6
P02790	Hemopexin	HEMO	51,676.37	6.55	16.45	5
Q9UQ35	Serine/arginine repetitive matrix protein 2	SRRM2	299,615.1	12.05	7.27	14
P62937	Peptidyl-prolylcis-trans isomerase A	PPIA	18,012.49	7.68	24.24	4
P84098	Ribosomal protein L19	RL19	23,465.97	11.48	13.47	2
Q9NZI8	Insulin-like growth factor 2 mRNA-binding protein 1	IF2B1	63,480.59	9.26	22.18	10
Q9HCE1	Putative helicase MOV-10	MOV10	113,671.3	9	14.86	11
Q15717	ELAV-like protein 1	ELAV1	36,091.88	9.23	28.22	7
Q00325	Phosphate carrier protein, mitochondrial	MPCP	40,094.86	9.45	15.75	6
Q07666	KH domain-containing, RNA-binding, signal transduction-associated protein 1	KHDR1	48,227.34	8.73	9.71	3
Q01658	Protein Dr1	NC2B	19,443.66	4.69	32.95	4
P09661	U2 small nuclear ribonucleoprotein A'	RU2A	28,415.57	8.71	26.27	5
P13010	X-ray repair cross-complementing protein 5	XRCC5	82,704.54	5.55	5.05	3
P36957	Dihydrolipoyllysine-residue succinyltransferase component of 2-oxoglutarate dehydrogenase complex, mitochondrial	ODO2	48,755.31	9.1	12.58	5
P22061	Protein-L-isoaspartate O-methyltransferase	PIMT	24,636.38	6.7	19.23	6
P35659	Protein DEK	DEK	42,674.28	8.69	12.00	4
P02765	α-2-HS-glycoprotein	FETUA	39,324.68	5.43	13.90	5
Q9UKM9	RNA-binding protein Raly	RALY	32,463.17	9.2	24.89	6
O43809	Cleavage and polyadenylation-specificity factor subunit 5	CPSF5	26,227.29	8.85	28.00	4
Q08431	Lactadherin	MFGM	43,122.99	8.47	16.80	5
P35637	RNA-binding protein FUS	FUS	53,425.84	9.4	11.57	4
P22087	rRNA 2'-O-methyltransferase fibrillarin	FBRL	33,784.22	10.18	19.31	4
O75475	PC4 and SFRS1-interacting protein	PSIP1	60,103.24	9.15	11.32	5
P40926	Malate dehydrogenase, mitochondrial	MDHM	35,503.28	8.92	20.12	6
P62826	GTP-binding nuclear protein Ran	RAN	24,423.11	7.01	15.02	4
Q9Y3Y2	Chromatin target of PRMT1 protein	CHTOP	26,396.57	12.24	19.35	4
Q9NR30	Nucleolar RNA helicase 2	DDX21	87,344.4	9.32	10.09	6
P84090	Enhancer of rudimentary homolog	ERH	12,258.94	5.62	37.50	3
Q9Y383	Putative RNA-binding protein Luc7-like 2	LC7L2	46,513.9	10.02	14.54	5
P55769	NHP2-like protein 1	NH2L1	14,173.55	8.72	26.52	3
P42167	Lamina-associated polypeptide 2, isoforms β/γ	LAP2B	50,670.26	9.39	12.56	4
P63162	Small nuclear ribonucleoprotein-associated protein N	RSMN	24,614.04	11.2	17.16	3
P57721	Poly(rC)-binding protein 3	PCBP3	39,465.25	8.22	12.47	3
P02763	α-1-acid glycoprotein 1	A1AG1	23,511.56	4.93	17.41	3
P26368	Splicing factor U2AF 65 kDa subunit	U2AF2	53,500.98	9.19	10.11	3
Q6PKG0	La-related protein 1	LARP1	123,510.3	8.91	6.57	5
P11182	Lipoamideacyltransferase component of branched-chain α-keto acid dehydrogenase complex, mitochondrial	ODB2	53,487.07	8.71	11.62	4
P04637	Cellular tumor antigen p53	P53	43,653.18	6.33	5.34	2
P14174	Macrophage migration inhibitory factor	MIF	12,476.3	7.73	17.39	2
P38919	Eukaryotic initiation factor 4A-III	IF4A3	46,871.03	6.3	13.14	5
Q07021	Complement component 1 Q subcomponent-binding protein, mitochondrial	C1QBP	31,362.24	4.74	12.06	2
P12277	Creatine kinase B-type	KCRB	42,644.28	5.34	15.22	4
P46013	Antigen KI-67	KI67	358,693.7	9.49	2.18	3
P00450	Ceruloplasmin	CERU	122,205.2	5.44	3.91	3
Q92900	Regulator of nonsense transcripts 1	RENT1	124,345.3	6.18	3.81	4
O43175	D-3-phosphoglycerate dehydrogenase	SERA	56,650.5	6.29	5.63	3
P06454	Prothymosin α	PTMA	12,202.96	3.66	35.51	3
Q16576	Histone-binding protein RBBP7	RBBP7	47,820.08	4.89	5.53	2
P61326	Protein magonashi homolog	MGN	17,163.62	5.74	21.23	2
P02774	Vitamin D-binding protein	VTDB	52,963.65	5.4	11.13	3
O75955	Flotillin-1	FLOT1	47,355.28	7.08	11.48	4
Q9Y230	RuvB-like 2	RUVB2	51,156.57	5.49	7.34	3
P63167	Dynein light chain 1, cytoplasmic	DYL1	10,365.88	6.89	24.72	2
P18754	Regulator of chromosome condensation	RCC1	44,969.02	7.18	8.07	2
O43143	Pre-mRNA-splicing factor ATP-dependent RNA helicase DHX15	DHX15	90,932.83	7.12	2.77	2
P20042	Eukaryotic translation initiation factor 2 subunit 2	IF2B	38,388.41	5.6	6.61	2
Q06787	Fragile X mental retardation 1, isoform CRA_e	FMR1	71,174.48	7	8.95	5
P27824	Calnexin	CALX	67,568.3	4.46	4.56	2
P51114	Fragile X mental retardation syndrome-related protein 1	FXR1	69,720.79	5.84	3.67	1
Q9NY12	H/ACA ribonucleoprotein complex subunit 1	GAR1	22,347.88	10.91	12.44	2
P59190	Ras-related protein Rab-15	RAB15	24,375.19	5.53	10.38	2
Q9NZ01	Very-long-chain enoyl-CoA reductase	TECR	36,010.78	9.50	5.84	2
P78527	DNA-dependent protein kinase catalytic subunit	PRKDC	469,088.8	6.75	0.56	2
P22234	Multifunctional protein ADE2	PUR6	47,079.22	6.94	6.30	2
O14893	Gem-associated protein 2	GEMI2	31,585.12	5.43	12.14	2
Q15388	Mitochondrial import receptor subunit TOM20 homolog	TOM20	16,297.88	8.81	22.76	2
P61604	10 kDa heat shock protein, mitochondrial	CH10	10,931.69	8.89	46.81	2
Q13263	Transcription intermediary factor 1-β	TIF1B	88,549.66	5.52	2.39	2
Q04837	Single-stranded DNA-binding protein, mitochondrial	SSBP	17,259.67	9.59	15.54	2
Q09161	Nuclear cap-binding protein subunit 1	NCBP1	91,839.44	5.99	4.05	2
Q9P035	Very-long-chain (3R)-3-hydroxyacyl-CoA dehydratase 3	HACD3	43,159.55	9.04	8.84	2
P04003	C4b-binding protein α chain	C4BPA	67,033.19	7.15	1.84	1
P01042	Kininogen-1	KNG1	71,957.38	6.34	2.95	1
Q96IX5	Up-regulated during skeletal muscle growth protein 5	USMG5	6457.57	9.78	25.86	1
P04004	Vitronectin	VTNC	54,305.59	5.55	2.51	1
Q9NUD5	Zinc finger CCHC domain-containing protein 3	ZCHC3	43,618.48	8.86	3.96	1
P85037	Forkhead box protein K1	FOXK1	75,457.34	9.41	2.59	2
Q96SB3	Neurabin-2	NEB2	89,192.07	4.91	6.12	3
P35232	Prohibitin	PHB	29,804.1	5.57	8.54	2
P02749	β-2-glycoprotein 1	APOH	38,298.16	8.34	8.70	2
Q13838	Spliceosome RNA helicase DDX39B	DX39B	48,991.33	5.44	7.49	1
O76021	Ribosomal L1 domain-containing protein 1	RL1D1	54,972.52	10.13	2.56	1
O43707	α-actinin-4	ACTN4	104,854	5.27	1.32	1
Q9UN86	RasGTPase-activating protein-binding protein 2	G3BP2	54,121.13	5.41	5.39	2
Q969G3	SWI/SNF-related matrix-associated actin-dependent regulator of chromatin subfamily E member 1	SMCE1	46,649.42	4.84	7.34	1
Q8WXI9	Transcriptional repressor p66-β	P66B	65,260.72	9.73	1.85	1
Q15287	RNA-binding protein with serine-rich domain 1	RNPS1	34,208.24	11.85	7.11	1
O96019	Actin-like protein 6A	ACL6A	47,460.97	5.39	3.03	1
P40425	Pre-B-cell leukemia transcription factor 1	PBX2	45,881.29	7.18	10.79	1
Q5UIP0	Telomere-associated protein RIF1	RIF1	274,465.6	5.39	0.57	1
P49006	MARCKS-related protein	MRP	19,528.8	4.65	7.69	1
P25788	Proteasome subunit α type-3	PSA3	28,433.23	5.19	4.71	1
Q6PJP8	DNA cross-link repair 1A protein	DCR1A	116,399.6	8.24	1.06	1
P62995	Transformer-2 protein homolog β	TRA2B	33,665.68	11.25	11.02	1
Q9Y2Q9	28S ribosomal protein S28, mitochondrial	RT28	20,842.78	9.22	6.29	1
P13797	Plastin-3	PLST	70,811.02	5.41	1.62	1
Q13428	Treacle protein	TCOF	152,106	9.06	0.99	1
Q96CT7	Coiled-coil domain-containing protein 124	CC124	25,835.24	9.54	9.87	2
Q9UHX1	Poly(U)-binding-splicing factor PUF60	PUF60	59,875.47	5.19	2.86	1
P55072	Transitional endoplasmic reticulum ATPase	TERA	89,321.8	5.14	2.11	1
P08579	U2 small nuclear ribonucleoprotein B''	RU2B	25,486.33	9.72	11.56	2
O75533	Splicing factor 3B subunit 1	SF3B1	145,830.4	6.65	3.53	4
P02647	Apolipoprotein A-I	APOA1	30,777.83	5.56	62.92	20
Q07955	Serine/arginine-rich-splicing factor 1	SRSF1	27,744.58	10.37	37.15	11
P53999	Activated RNA polymerase II transcriptional coactivator p15	TCP4	14,395.34	9.6	60.63	8
Q15233	Non-POU domain-containing octamer-binding protein	NONO	54,231.54	9.01	28.87	14
P35611	α-Adducin	ADDA	80,955.14	5.6	21.04	11
Q16352	α-Internexin	AINX	55,390.65	5.34	47.29	20
P52272	Heterogeneous nuclear ribonucleoprotein M	HNRPM	77,515.53	8.84	43.42	29
O75165	DnaJ homolog subfamily C member 13	DJC13	254,414.9	6.31	6.69	18

### 2.3. Lysine Acetylation Modifications Identified in Some of Cellular Proteins Co-Immunoprecipitated with NP

**Figure 2:**
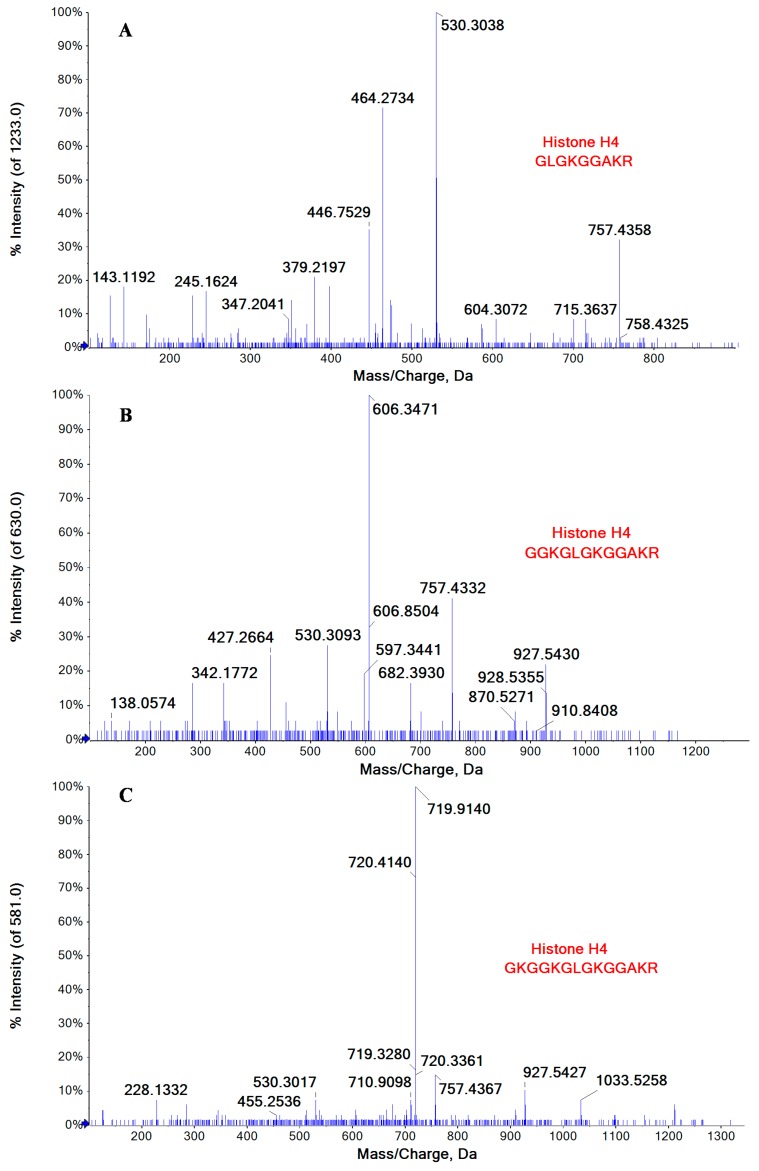
MS/MS spectra of three correlated peptides (**A**) GLGKGGAKR (10–18); (**B**) GGKGLGKGGAKR (7–18); and (**C**) GKGGKGLGKGGAKR (5–18) of Histone H4, in which all the lysine residues were acetylated.

Among the identified host proteins co-immunoprecipitated with NP, some were found to be acetylated at lysine residues, including the Histone H3 and H4 that were well-recognized as the chief protein components of chromatin. Figure 2 illustrated MS/MS spectra of three correlated peptides GLGKGGAKR (10–18), GGKGLGKGGAKR (7–18), and GKGGKGLGKGGAKR (5–18) of Histone H4, in which all the lysine residues in these peptides were acetylated. Histones play a key role in gene regulation, which could be affected by several kinds of posttranslational modifications (methylation, acetylation, phosphorylation, and so on) that alter their interaction with DNA and nuclear proteins. Influenza A virus vRNPs were reported to associate with vRNPs interact with histone tails to modulate the release of vRNPs from chromatin [18]. Besides Histone H3 and H4, six host proteins that were co-immunoprecipitated with NP were also observed to be acetylated at their lysine residues, as indicated in Table 2.

### 2.4. Bioinformatics Analysis

**Figure 3:**
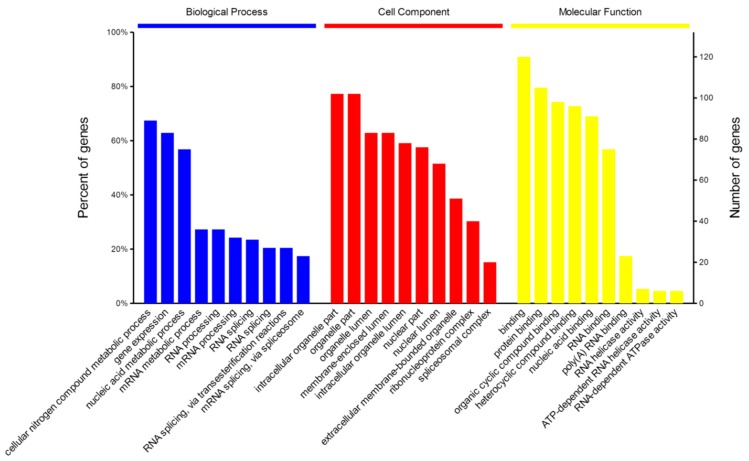
GO annotation of identified NP-related proteins in three categories: biological process (BP), cellular component (CC) and molecular function (MF).

The obtained protein data were analyzed using bioinformatics approaches, in an effort to extract information relevant to involved pathways. An overview of NP-related proteins in biological process (BP), cell component (CC), and molecular function (MF) categories by gene ontology (GO) analysis, respectively, was shown in Figure 3. In the BP analysis, the majority of identified proteins were classified into metabolic processes, especially in cellular nitrogen compound metabolic process and nucleic acid metabolic process. The CC analysis showed that most of identified protein belonged to organelle and nuclear component. Molecular functional classification of these proteins revealed that most were involved in protein binding, cyclic compound binding, and nucleic acid binding. The result from GO analysis indicated that these NP-related host proteins exhibited a wide variety of cellular distributions and functions, in accordance with the fact that NP, the structural component of the virus, participated in multiple indispensable activities via its interaction with the components of host cells [19,20,21].

**Table 2:** Identification of lysine acetylation modifications on host cellular proteins co-immunoprecipitated with NP.

Accession	Protein Name	Lysine-Acetylated Peptide	Residues in Protein
P62805	Histone H4	GK*GGK*GLGK*GGAK*R	5–18
		GGK*GLGK*GGAK*R	7–18
		GLGK*GGAK*R	10–18
Q5TEC6	Histone H3	K*STGGK*APR	10–18
		K*QLATK*AAR	19–27
Q15149	Plectin	IEQEK*AKLEQLFQDEVAK	2646–2663
P08670	Vimentin	ASLARLDLERK*VESLQEEIAFLK	213–235
Q9UHB6	LIM domain and actin-binding protein 1	STPAEDDSRDSQVK*	336–349
P15880	40S ribosomal protein S2	TK*SPYQEFTDHLVK	262–275
P62158	Calmodulin	HVMTNLGEK*LTDEEVDEMIR	108–127
Q86V81	THO complex subunit 4	ADK*MDMSLDDIIK	2–14

K* refers to acetylated lysine residue.

**Figure 4:**
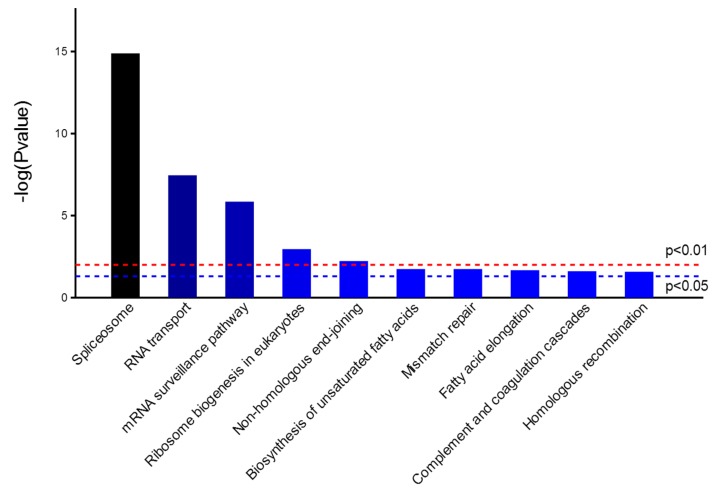
Distribution of enriched KEGG pathway. Columns refer to related pathways, which are colored with gradient colors from midnight blue (smaller *p*-value) to lighter blue (bigger *p*-value).

**Figure 5:**
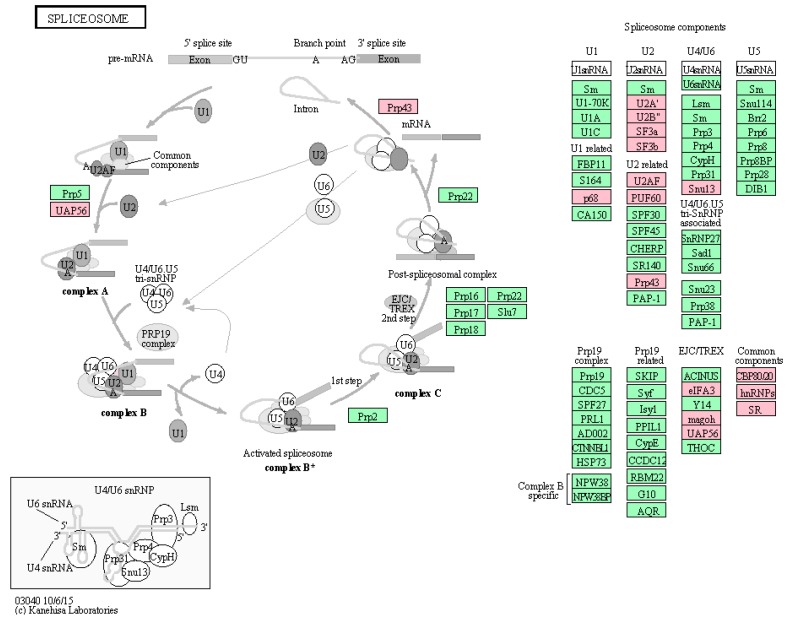
Significantly enriched spliceosome pathway. Up to seventeen proteins labeled in pink were identified by proteomics approach in the present study, which were co-immunoprecipitated with NP. Seventeen proteins displayed in pink color were listed as follows: P68; U2Aʹ; U2Bʺ; SF3a; SF3b; U2AF; PUF60; Prp43; Snu13; eIFA3; magoh; UAP56; CBP80/20(NCBP1); hnRNPs(RBMX,HNRNPM); SR(SRSF1,TRA2B). The green color represents other proteins in spliceosome pathway.

**Figure 6:**
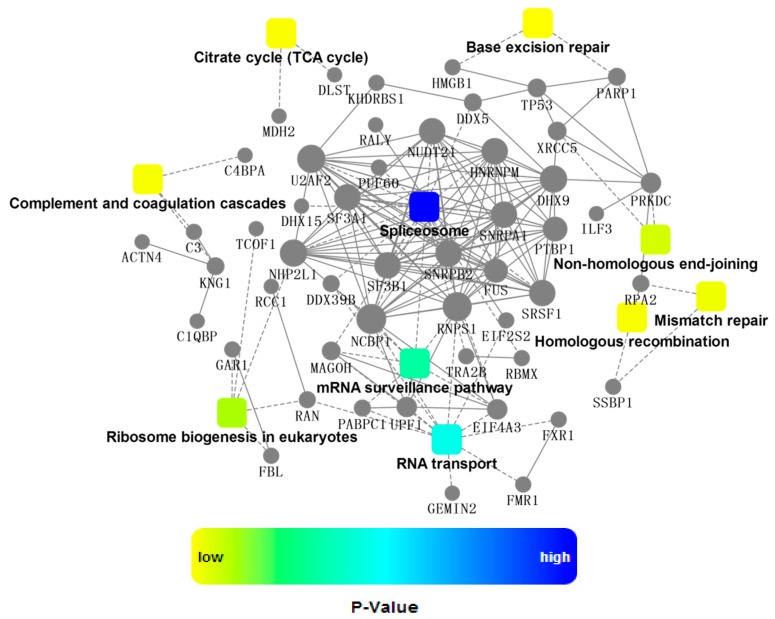
A network of protein-protein interaction (PPI). The PPI analysis was based on fold change of gene/protein, protein-protein interaction, KEGG pathway enrichment and biological process enrichment. Circle nodes refer to genes/proteins. Rectangle refers to KEGG pathway or biological process, which were colored with gradient color from yellow (smaller *p*-value) to blue (bigger *p*-value).

Next, KEGG analysis revealed that the most active pathways involved were those related to RNA metabolism that was well concordant with the major functions of NP (Figure 4). Among them, spliceosome was the most significantly enriched pathway, which contained seventeen proteins co-immunoprecipitated with NP, as shown in Figure 5. NP was supposed to interact with spliceosome in host cells, interfering with the maturity of mRNA from pre-mRNA. For example, interaction of NP with a cellular splicing factor, UAP56, resulted in enhanced influenza virus RNA synthesis. UAP56 was found to bind to the N-terminal region of NP, a domain essential for RNA binding, facilitating the formation of complexes between NP and RNA [22]. In addition, other processing and splicing factors, including heterogeneous nuclear ribonucleoproteins (hn-RNPs) and serine–arginine-rich (SR) proteins, were characterized as interacting partners with viral NP. hnRNPs are supposed to function to prevent the folding of pre-mRNA and to export mRNA out of the nucleus, while SR proteins can act as splicing enhancers by stabilizing the spliceosome assembly. SRSF1, one of SR proteins identified by proteomics approaches in this study, was found to be involved in interaction between H9N2 virus and infected human cells [23], which was further confirmed by Western blotting analysis as indicated in Figure S2 in supplementary files. To further extract relevant information from the identified protein data, a more comprehensive bioinformatics analysis of the proteomics data was performed using Cytoscape, a powerful tool for integrating protein-protein interaction (PPI) networks into a unified conceptual framework. Again, PPI analysis identified spliceosome as the most significantly enriched pathways indicated in Figure 6.

Throughout infection process, influenza viruses hijack a variety of host biochemical machineries. For example, influenza viruses rely on host spliceosome to generate specific spliced influenza virus products during their replication cycles. As a necessary step for viral replication, splicing appears to be much important, especially for “simple” organisms with very small genome such as influenza. Even though several spliced transcripts of NS and M segments have been well characterized, the molecular mechanism underlined is still not fully understood. Therefore, efforts should be made to better understand the fine regulation mechanisms of splicing of viral segments, with respect to viral replication, host range, and pathogenicity.

## 3. Experimental Section

### 3.1. Chemicals and Materials

Sequencing-grade TPCK-modified trypsin was purchased from Promega (Madison, WI, USA). HPLC grade ACN and methanol were from Fisher (Fairlawn, NY, USA). Pierce crosslink magnetic IP/co-IP kit, as well as HRP-Conjugated Goat anti-Rabbit IgG (H + L), was from Pierce (Rockford, IL, USA). Rabbit anti-β-actin antibody was purchased from Cell Signaling Technology (Beverly, MA, USA). Lipofactamine 2000 was obtained from Life Technologies (Carlsbad, CA, USA). Bradford protein quantification reagent was purchased from Bio-Rad (Hercules, CA, USA). 1.5 mL 10KD ultrafiltration centrifuge tubes were from Millipore (Bedford, MA, USA). Rabbit anti-nucleoprotein monoclonal antibody was from Sino Biological (Beijing, China). Anti-SRSF1 polyclonal antibody was from Santa Cruz Biotechnology (Dallas, TX, USA). High glucose DMEM medium and fetal bovine serum (FBS) were purchased from Hyclone (Logan, UT, USA). Endo-free plasmid maxi kit was a product from OMEGA Bio-Tec (Norcross, GA, USA). ECL chemiluminescent reagents were from Thermo Scientific (Rockford, IL, USA). Ammonium bicarbonate, dithiothreitol (DTT), and iodoacetamide (IAA) were purchased from Bio-Rad (Hercules, CA, USA). All the other chemicals were purchased from Sigma-Aldrich (St. Louis, MO, USA). Ultra-pure water was prepared by a MilliQ water purification system (Millpore, Bedford, MA, USA).

### 3.2. Plasmid Construction, Amplification and Transfection

The pCMV-NP plasmid was constructed by Sino Biological Inc (Beijing, China). The full length NP sequence (Influenza A virus H7N9 (A/shanghai/1/2013)) was inserted into pCMV plasmid through two restriction sites (KpnI and XbaI). The pCMV-NP plasmid was transformed into E.coli DH5α, of which the positive clone was screened on LB agar plate containing 60 μg/mL Ampicillin. Then, the selected clone was amplified with shaking (200 rpm) in LB media supplemented with 60 μg/mL Ampicillin at 37 °C overnight and the plasmids were extracted using endo-free plasmid maxi kit (OMEGA). To confirm the sequence accuracy of cloned NP, the pCMV-NP plasmid was sequenced using a pair of primers as follows: TAATACGACTCACTATAGGG (forward), TAGAAGGCACAGTCGAGG (reverse).

HEK293T cells were cultured in high glucose DMEM medium containing 10% fetal bovine serum at 37 °C in a CO_2_ incubator. Until the cell density reached 80%–90%, the pCMV-NP plasmid containing full length gene of nucleoprotein from influenza A H7N9 (A/shanghai/1/2013) was transfected into HEK293T cells cultured in 10 cm dishes using Lipofactamine 2000 following the manufacturer’s protocol. As a negative control, an empty pCMV plasmid was transfected into HEK293T cells under the same conditions as above. All the transfected cells were incubated for 36 h.

### 3.3. Co-Immunoprecipitation

The crosslink magnetic IP/Co-IP kit from pierce was used to capture nucleoprotein-binding cellular proteins by co-immunoprecipitation (co-IP) according to the manufacturer’s instruction. Briefly, the beads were pre-washed two times with 1X Modified Coupling Buffer and then bound with rabbit anti-nucleoprotein monoclonal antibody for 15 min. After washed three times with 1X Modified Coupling Buffer, the beads were covalently coupled with the bound antibody by disuccinimidylsuberate (DSS) for 30 min. The crosslinked beads were washed three times with Elution Buffer to remove unbound antibody in the reaction mixture, followed by two washes with IP Lysis/Wash buffer.

The transfected cells were lysed in IP Lysis/Wash buffer (pH 7.4, 25 mM Tris, 150 mM NaCl, 1 mM EDTA, 1% NP40, 5% glycerol). The lysates were cleared by centrifugation and adjusted to identical concentration by IP Lysis/Wash buffer after Bradford protein quantification, from which small aliquots were removed for Western blotting analysis. The antibody-crosslinked beads were added into the lysates and incubated overnight at 4 °C. The beads were collected by magnetic force and the supernatants were transferred into new vials for Western blotting analysis. After washed several times with IP Lysis/Wash Buffer and one time with ultrapure water, the beads were incubated with Elution Buffer, from which the antigen (nucleoprotein), as well as the co-precipitated cellular proteins, was eluted. A negative control IP was performed as above, except that lysates of HEK293T cells transfected with an empty pCMV plasmid was used. To neutralize the low pH, add 10 μL of Neutralization Buffer for each 100 μL of eluate for each sample. All samples were stored at −80 °C, from which small aliquots were removed for Western Blotting analysis.

### 3.4. SDS-PAGE and Western Blotting

Samples were subjected to electrophoresis in 12% Tris-glycine-SDS polyacrylamide gel using a Mini-Cell system (Bio-Rad, Hercules, CA, USA). Gels were electrophoretically transferred onto polyvinylidene fluoride (PVDF) membranes (0.45 μm pore size). The blotted membranes were blocked with 5% nonfat dry milk in a buffer (25 mM Tris, pH 7.5, 150 mM NaCl, and 0.05% Tween 20) for 2 h at room temperature, followed by incubation with the diluted primary antibody against NP for 4 h at room temperature. After washing for 10 min in TBST solution, membranes were incubated with properly diluted secondary antibody conjugated with horseradish peroxidase for 2 h at room temperature. Western signals were developed using ECL chemiluminescent reagents from Thermo Scientific (Waltham, MA, USA).

### 3.5. Filter-Aided Buffer Exchange and Trypsin Digestion

Prior to in-solution tryptic digestion, the samples were subject to buffer exchange as previously described [24]. Briefly, samples were diluted with equal volume of Buffer 1 (8 M urea, 0.1 M Tris-HCl, pH 8.0) and transferred into 1.5 mL 10KD ultrafiltration centrifuge tubes. After centrifugation (12,000 rpm, 20 min, 4 °C), the concentrate was diluted with 200 μL of Buffer 1 and the ultrafiltration device was centrifuged. This buffer exchange was repeated for additional two times. Then, the sample was diluted with 90 µL Buffer 1 containing 10 mM DTT and incubated at 37 °C for 1 h, followed by centrifugation (12,000 rpm, 10 min, 4 °C). The alkylation reaction was carried out by adding 90 µL Buffer 1 containing 50 mM iodoacetamide in the dark at room temperature for 15 min. After centrifugation (12,000 rpm, 10 min, 4 °C), the protein sample was washed three times (one wash with Buffer 1 and two washes with 50 mM ammonium bicarbonate solution). TPCK-modified sequencing-grade trypsin was added at an enzyme/protein ratio of 1:100. Digestion was performed at 37 °C for at least 15 h and stopped by adding 10% formic acid to a final concentration of 1%. The tryptic digests were collected by centrifugation andpurified over C18 Ziptips. The desalted digests were freeze-dried and kept at −80 °C.

### 3.6. Nano-LC-MS/MS and Data Processing

The desalted peptides were re-solubilized in 10 μL of 0.1% (vol/vol) trifluoroacetic acid. The tryptic peptide sample was loaded onto a peptide trap column, then separated by a C18 capillary column (ChromXP, Eksigent Technologies, 150 mm × 75 μm × 3.0 μm, Silicon valley, San Francisco, CA, USA) at 300 nL/min delivered by an Eksigent nanoLC pump (Silicon valley). The elution gradient was run using mobile phase A (2% acetonitrile/0.1% formic acid) and B (98% acetonitrile/0.1% formic acid) from 0 to 60 min with 5%–30% B followed by 60–75 min with 28%–42% B and 75–85 min with 42%–85% B. A TripleTOF 5600+ mass spectrometer coupled with a nanospray source was used to analyze peptides eluted from capillary C18 chromatography. Information Dependent Acquisition was chosen to perform MS/MS experiments, wherein the switch criteria were as follows: the range of *m*/*z* is 350–1250 *m*/*z*; the number of charged ions is 2–5; the collision energy is applied in the mode of Rolling Collision Energy.

The collected data files (.wiff) were transferred to the data processing workstation. MS data analysis software ProteinPilot 5.0 (AB Sciex, Framingham, MA, USA) was used for protein database searching against SwissProt database. Parameters were set as follows: protease was chosen as Trypsin; alkylation of Cys by iodoacetamide is chosen; biological modifications were chosen as ID Focus.

### 3.7. Bioinformatics Analysis

The multi-omics data analysis tool, OmicsBean, was used to analyze the obtained proteomics data (http://www.omicsbean.com:88/), in which distributions in biological functions, subcellular locations and molecular functions were assigned to each protein based on Gene Ontology (GO) categories. The Kyoto Encyclopedia of Genes and Genomes (KEGG) pathway analysis was performed in order to enrich high-level functions in the defined biological systems. Protein-protein interaction (PPI) analysis was using Cytoscape software [25], in which confidence cutoff of 400 was used: interactions with bigger confident score were show as solid lines between genes/proteins, otherwise in dashed lines.

## 4. Conclusions

Avian influenza A viruses are serious veterinary pathogens that normally circulate among avian populations, causing substantial economic impacts. Some strains of avian influenza A viruses, such as H5N1, H9N2, and recently reported H7N9, have been occasionally found to adapt to humans from other species. In order to replicate efficiently in the new host, influenza viruses have to interact with a variety of host factors. The present study identified a variety of host proteins that might interact with H7N9 nucleoprotein expressed in human HEK293T cells, using a proteomics approach. Bioinformatics analysis suggested a role for spliceosome pathway in host response to nucleoprotein expression, increasing our emerging knowledge of host proteins that might be involved in influenza virus replication activities.

## Supplementary Materials

Supplementary materials can be found at http://www.mdpi.com/1422-0067/16/11/25934/s1.

## References

[1] Peiris J.S., Yu W.C., Leung C.W., Cheung C.Y., Ng W.F., Nicholls J.M., Ng T.K., Chan K.H., Lai S.T., Lim W.L. (2004). Re-emergence of fatal human influenza A subtype H5N1 disease. Lancet.

[2] Peiris M., Yuen K.Y., Leung C.W., Chan K.H., Ip P.L., Lai R.W., Orr W.K., Shortridge K.F. (1999). Human infection with influenza H9N2. Lancet.

[3] Li K.S., Guan Y., Wang J., Smith G.J., Xu K.M., Duan L., Rahardjo A.P., Puthavathana P., Buranathai C., Nguyen T.D. (2004). Genesis of a highly pathogenic and potentially pandemic H5N1 influenza virus in eastern Asia. Nature.

[4] Wu S., Wu F., He J. (2013). Emerging risk of H7N9 influenza in China. Lancet.

[5] Gao R., Cao B., Hu Y., Feng Z., Wang D., Hu W., Chen J., Jie Z., Qiu H., Xu K. (2013). Human infection with a novel avian-origin influenza A (H7N9) virus. N. Engl. J. Med..

[6] Pan H., Zhang X., Hu J., Chen J., Pan Q., Teng Z., Zheng Y., Mao S., Zhang H., King C.C. (2015). A case report of avian influenza H7N9 killing a young doctor in Shanghai, China. BMC Infect. Dis..

[7] Palese P., Shaw M.L. (2007). Fields virology. Orthomyxoviridae: The Viruses and Their Replication.

[8] Chenavas S., Crépin T., Delmas B., Ruigrok R.W., Slama-Schwok A. (2013). Influenza virus nucleoprotein: structure, RNA binding, oligomerization and antiviral drug target. Future Microbiol..

[9] Vreede F.T., Brownlee G.G. (2007). Influenza virion-derived viral ribonucleoproteins synthesize both mRNA and cRNA *in vitro*. J. Virol..

[10] Newcomb L.L., Kuo R.L., Ye Q., Jiang Y., Tao Y.J., Krug R.M. (2009). Interaction of the influenza a virus nucleocapsid protein with the viral RNA polymerase potentiates unprimed viral RNA replication. J. Virol..

[11] Sharma S., Mayank A.K., Nailwal H., Tripathi S., Patel J.R., Bowzard J.B., Gaur P., Donis R.O., Katz J.M., Cox N.J. (2014). Influenza A viral nucleoprotein interacts with cytoskeleton scaffolding protein α-actinin-4 for viral replication. FEBS J..

[12] Generous A., Thorson M., Barcus J., Jacher J., Busch M., Sleister H. (2014). Identification of putative interactions between swine and human influenza A virus nucleoprotein and human host proteins. Virol. J..

[13] Mayer D., Molawi K., Martínez-Sobrido L., Ghanem A., Thomas S., Baginsky S., Grossmann J., García-Sastre A., Schwemmle M. (2007). Identification of cellular interaction partners of the influenza virus ribonucleoprotein complex and polymerase complex using proteomic-based approaches. J. Proteome Res..

[14] Kao R.Y., Yang D., Lau L.S., Tsui W.H., Hu L., Dai J., Chan M.P., Chan C.M., Wang P., Zheng B.J. (2010). Identification of influenza A nucleoprotein as an antiviral target. Nat. Biotechnol..

[15] Chen G.W., Chang S.C., Mok C.K., Lo Y.L., Kung Y.N., Huang J.H., Shih Y.H., Wang J.Y., Chiang C., Chen C.J. (2006). Genomic signatures of human *versus* avian influenza A viruses. Emerg. Infect. Dis..

[16] Pan C., Cheung B., Tan S., Li C., Li L., Liu S., Jiang S. (2010). Genomic signature and mutation trend analysis of pandemic (H1N1) 2009 influenza A virus. PLoS ONE.

[17] Mänz B., Schwemmle M., Brunotte L. (2013). Adaptation of avian influenza A virus polymerase in mammals to overcome the host species barrier. J. Virol..

[18] Garcia-Robles I., Akarsu H., Müller C.W., Ruigrok R.W., Baudin F. (2005). Interaction of influenza virus proteins with nucleosomes. Virology.

[19] Portela A., Digard P. (2002). The influenza virus nucleoprotein: A multifunctional RNA-binding protein pivotal to virus replication. J. Gen. Virol..

[20] Nailwal H., Sharma S., Mayank A.K., Lal S.K. (2015). The nucleoprotein of influenza A virus induces p53 signaling and apoptosis via attenuation of host ubiquitin ligase RNF43. Cell. Death Dis..

[21] Elton D., Simpson-Holley M., Archer K., Medcalf L., Hallam R., McCauley J., Digard P. (2001). Interaction of the influenza virus nucleoprotein with the cellular CRM1-mediated nuclear export pathway. J. Virol..

[22] Momose F., Basler C.F., O’Neill R.E., Iwamatsu A., Palese P., Nagata K. (2001). Cellular splicing factor RAF-2p48/NPI-5/BAT1/UAP56 interacts with the influenza virus nucleoprotein and enhances viral RNA synthesis. J. Virol..

[23] Liu N., Song W., Wang P., Lee K., Chan W., Chen H., Cai Z. (2008). Proteomics analysis of differential expression of cellular proteins in response to avian H9N2 virus infection in human cells. Proteomics.

[24] Wiśniewski J.R., Zougman A., Nagaraj N., Mann M. (2009). Universal sample preparation method for proteome analysis. Nat. Methods.

[25] Shannon P., Markiel A., Ozier O., Baliga N.S., Wang J.T., Ramage D., Amin N., Schwikowski B., Ideker T. (2003). Cytoscape: A software environment for integrated models of biomolecular interaction networks. Genome Res..

