# Supplementary material for: Proteomics Analysis of Cellular Proteins Co-Immunoprecipitated with Nucleoprotein of Influenza A Virus (H7N9)
Source: Int J Mol Sci. 2015 Oct 30;16(11):25982–98. doi: 10.3390/ijms161125934 (PMC4661793; doi:10.3390/ijms161125934)
Supplement: Supplementary file 1 [file ijms-16-25934-s001.zip › ijms-98921-Supplementary Information/ijms-98921-Figures S1 and S2.pdf]

International Journal of
Molecular Sciences

MDPI

Supplementary Information

primers: TAATACGACTCACTATAGGG (forward),

TAGAAGGCACAGTCGAGG (reverse).

ATGGCCTCTCAAGGCACTAAACGGTCTTATGAACAGATGGAAACTGGTGGAGAACGCCAGAATGCCACT
GAAATCAGAGCATCCGTCGGAAGAATGGTTAGTGGAATTGGACGATTCTACATCCAAATGTGCACCGAAC
TCAAACTCAGTGATAATGAGGGACGGTTGATCCAAAACAGCATAACAATAGAGAGAATGGTGCTCTCTG
CTTTTGACGAAAGGAGAAATAGATACCTTGAAGAACATCCCAGTGCGGGGAAAGATCCTAAGAAAACTG
GAGGACCTATATACAGGAGACGAGACGGAAAGTGGGTGAGAGAACTCATCCTTTATGACAAAGAAGAAA
TAAGGCGAATCTGGCGCCAAGCTAATAATGGTGAAGATGCAACGGCTGGTCTGACTCACCTGATGATCTG
GCATTCCAATTTGAATGATGCAACTTATCAGAGGACAAGAGCTCTTGTTCGCACCGGAATGGATCCCAGG
ATGTGCTCTCTGATGCAAGGTTCAACTCTCCCTAGGAGGTCTGGAGCCGCAGGTGCTGCAGTCAAAGGA
ATTGGAACAATGGTGATGGAATTGATCAGAATGATCAAACGTGGGATCAATGATCGGAACTTCTGGAGGG
GTGAGAATGGACGAAGAACAAGGATTGCTTATGAGAGAATGTGCAACATTCTCAAAGGAAAATTTCAAA
CAGCTGCACAAAGAGCAATGGTGGATCAAGTGAGAGAAAGCCGGAACCCAGGAAATGCTGAGATCGAA
GATCTCATCTTTCTGGCACGGTCTGCACTCATATTGAGAGGGTCAGTTGCTCACAAATCTTGTCTGCCTGC
CTGTGTGTATGGACTTGCCGTAGCCAGTGGGTACGACTTTGAAAGGGAGGGATACTCTCTAGTCGGAATA
GACCCTTTCAGACTGCTTCAAAACAGCCAAGTGTTCAGCCTAATCAGACCAAATGAGAATCCAGCACAC
AAGAGTCAACTGGTGTGGATGGCATGCCATTCTGCCGCATTTGAAGATCTAAGAGTATCAAGCTTCATCA
GAGGGACGAGGATGGTCCCAAGAGGGCAGCTTTCCACTAGAGGAGTTCAAATTGCTTCCAATGAAAATA
TCGAGGCTATGGAGTCAAATACACTTGAACTGAGAAGCAGGTACTGGGCCATAAGGACCAGAAGTGGAG
GAAACACCAATCAACAGAGGGCATCTGCGGGCCAAATCAGCATACAACCTACGTTCTCAGTACAGAGAA
ATCTCCCTTTTGAGAGAGCAACCATTATGGCAGCATTCACTGGGAATACAGAGGGGAGAACATCTGACAT
GAGGACCGAAATCATAAGGATGATGGAAAGTGCAAGACCAGAAGATGTGTCTTTCCAGGGGCGGGGAG
TCTTCGAGCTCTCGGACGAAAAGGCAACGAACCCGATCGTGCCTTCCTTTGACATGAATAATGAAGGATC
TTATTTCTTCGGAGACAATGCCGAAGAGTACGATAACTAA

Figure S1. Sequencing result of pCMV-NP plasmid used in this study.

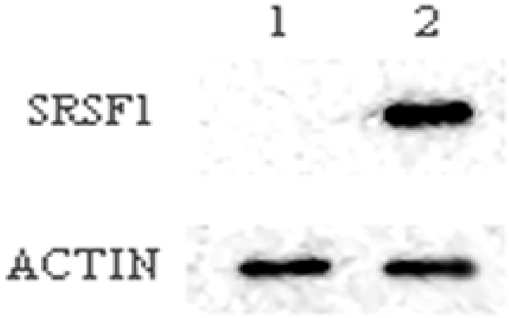

Figure S2. Western blot analysis of SRSF1 that was immunoprecipitated with NP. β-actin was used as a loading control. 1: empty pCMV; 2: pCMV-NP.
